# Hypoxia-associated genes and metabolic abnormalities in peripheral blood mononuclear cells of type 1 diabetes mellitus patients

**DOI:** 10.1186/s41065-025-00537-x

**Published:** 2025-08-21

**Authors:** Wen-biao Ma, Xue-ying Wang, Yuan-yuan Zuo

**Affiliations:** 1 Department of Emergency Medicine Center, The Second Qilu Hospital of Shandong University, No. 247 Beiyuan Street, Tianqiao District, Jinan, Shandong 250033 China; 2 Department of Clinical Laboratory, Shandong Provincial Hospital Affiliated to Shandong First Medical University, Jinan, Shandong 250021 China; 3 Department of Operating Room, Shandong Provincial Hospital Affiliated to Shandong First Medical University, Jinan, Shandong 250021 China

**Keywords:** Type 1 diabetes mellitus, Peripheral blood mononuclear cells, Differentially expressed genes, Hub genes, Hypoxia

## Abstract

**Background:**

Type 1 diabetes mellitus (T1DM) is a chronic autoimmune disorder characterized by insulin deficiency, which causes hyperglycemia and systemic metabolic dysregulation.

**Methods:**

In this study, we used the gene expression dataset GSE156035 to identify differentially expressed genes (DEGs) between healthy controls and patients with T1DM. Functional enrichment analysis, Gene Ontology analysis, and protein–protein interaction network analysis were employed to identify hub genes.

**Results:**

We observed significant upregulation and downregulation of DEGs. Upregulated genes were primarily involved in TGF-beta signaling and retinol metabolism, while downregulated genes were associated with MAPK signaling and circadian rhythm pathways. Crucial cellular processes, such as neutrophil activation, defense response to fungi, and neuron differentiation, were highlighted. Hub genes, such as *FOS*, J*UNB*, *NR4A2*, and *DUSP1*, were identified and showed strong correlations with key signaling pathways. Additionally, elevated levels of angiogenesis, epithelial-mesenchymal transition, and hypoxia in T1DM were indicated, along with significant alterations in metabolite levels, including glucose, leucine, and phenylalanine, and their correlations with hub genes.

**Conclusion:**

These findings not only identify specific hub genes as key mediators connecting signaling pathways, biological processes, and metabolic changes but also provide novel insights into the pathophysiology of T1DM.

## Introduction

Type 1 diabetes mellitus (T1DM) is a chronic autoimmune disorder characterized by the destruction of pancreatic β-cells, which leads to insulin deficiency and hyperglycemia [1]. T1DM affects millions of individuals worldwide, imposing significant health and economic burdens due to its associated complications, such as neuropathy, retinopathy, and cardiovascular diseases [2]. While current management strategies mainly focus on insulin therapy and lifestyle modifications, these approaches fail to address the underlying pathological molecular mechanisms or prevent disease progression [3]. While several studies have investigated the genetic and environmental factors contributing to T1DM, the identification of key regulatory genes and their functional roles in disease pathogenesis remains limited [4]. Furthermore, the integration of gene expression data with pathway and metabolite analyses to reveal the complex interplay between molecular networks and clinical outcomes has not been extensively investigated [5]. Addressing these gaps is critical for developing targeted therapies and improving patient outcomes, underscoring the necessity of this study [6].

T1DM is characterized by dysregulated immune responses and metabolic disturbances, yet the underlying molecular mechanisms are not completely understood [7]. Previous studies have identified altered gene expression and disrupted signaling pathways in T1DM, including TGF-beta and MAPK signaling, as well as retinol metabolism, all of which are implicated in immune modulation and metabolic regulation [8–10]. Additionally, biological processes, such as neutrophil activation, epithelial-mesenchymal transition (EMT), and hypoxia, have been associated with T1DM pathophysiology [11–13]. However, the interplay between these pathways, key regulatory genes, and metabolic changes remains underexplored. This study addresses this gap by integrating differential gene expression analysis, functional enrichment, protein–protein interaction (PPI) network construction, and correlation analyses to identify specific hub genes (*FOS*, *JUNB*, and *NR4A2*) that connect signaling pathways, biological processes, and metabolite dysregulation. These findings not only elucidate the molecular mechanisms of T1DM but also emphasize the potential of these hub genes as biomarkers and therapeutic targets, thereby providing novel insights into disease management.

This study employed a comprehensive bioinformatics approach to dissect the molecular mechanisms underlying T1DM by integrating gene expression analysis, functional enrichment, PPI network construction, and correlation studies. Leveraging the gene expression dataset GSE156035, differentially expressed genes (DEGs) between healthy controls (HCs) and patients with T1DM, and the biological significance of these genes were elucidated by functional enrichment analysis using Gene Ontology (GO) and KEGG pathways. The PPI network, which was constructed using the STRING database and visualized in Cytoscape, facilitated the identification of hub genes as central mediators in T1DM pathophysiology. Furthermore, this study examined correlations between hub genes and key signaling pathways, biological processes, and metabolites, thereby offering a holistic view of the molecular alterations associated with T1DM. The integration of these diverse analytical methods allows for a more thorough understanding of the interconnected pathways and processes altered in T1DM, with the ultimate goal of identifying potential biomarkers and therapeutic targets.

## Materials and Methods

### Data Acquisition and Preprocessing

The gene expression dataset used in this study was obtained from the Gene Expression Omnibus (GEO) repository under the accession number GSE156035. This dataset contains transcriptomic profiles of HCs and individuals with T1DM. The GSE156035 dataset was selected for this study based on its specific relevance to our research objectives. This dataset focuses on gene expression profiles in peripheral blood mononuclear cells (PBMCs) from patients with T1DM and healthy controls, which aligns with our goal of investigating molecular mechanisms in PBMCs—a key cell population involved in immune dysregulation and metabolic crosstalk in T1DM. Notably, GSE156035 includes well-characterized samples (20 T1DM patients and 20 healthy controls) with strict clinical phenotyping, ensuring the reliability of comparative analyses between groups. The raw data were processed and normalized using the Robust Multi-Array Averaging method from the “affy” package in R to ensure uniform expression levels across all samples. Data distribution was analyzed using boxplots to confirm consistent normalization across all samples.

### Identification of Differentially Expressed Genes

Differential gene expression analysis was performed using the “limma” package. A linear model was used to compare gene expression between the HC and T1DM groups, with a significance threshold set at an adjusted *p* value < 0.05 and |log2 fold-change|> 1. The resulting DEGs were visualized with a volcano plot, which highlighted genes that were significantly upregulated (red) and downregulated (blue) in the T1DM group relative to the HC group. A heatmap of the top DEGs was created to display gene expression patterns, with hierarchical clustering performed to group samples based on similarity in expression profiles.

### Functional Enrichment Analysis

To study the biological functions of the DEGs, GO and KEGG pathway enrichment analyses were conducted using the “clusterProfiler” package in R. The top enriched GO biological processes and KEGG pathways were identified for both upregulated and downregulated genes. The results were displayed using dot plots, where the dot size represented the count of genes involved in each pathway, and the color represented the level of statistical significance (− log10 adjusted *p* value).

### PPI Network Construction

The DEGs were further assessed by creating a PPI network using the STRING database (version 11.5). The PPI network was visualized using Cytoscape (version 3.8), which allowed for the identification of key functional modules and hub genes based on their connectivity degree within the network. Used a confidence score cutoff of > 0.7 (high confidence) to filter interactions. Employed CytoHubba with the Degree Centrality algorithm to identify hub genes (top 10% of nodes with highest connectivity). Genes with the highest degree of connectivity were considered potential key regulators in the pathophysiology of T1DM.

### Participants

A total of 10 patients were included in this study, comprising 5 patients with T1DM and 5 HCs. All samples were obtained from Shandong Provincial Hospital affiliated with Shandong First Medical University, with approval from the Ethics Committee for Biomedical Research Involving Humans (NSFC: NO. 2025–512). Patients with T1DM were selected based on strict criteria: 1) a confirmed diagnosis of T1DM for at least 1 year, 2) insulin dependence, 3) fasting C-peptide levels below 300 pmol/L, and 4) the presence of at least one autoantibody (GADA, IA-2A, ZnT8A, or IAA). Individuals with microvascular complications, acute or chronic infections, malignancies, or thyroid diseases were excluded from the study. HCs were individuals without diabetes or other autoimmune diseases. Blood samples were collected after an 8–12 h fast and subsequently used to isolate peripheral blood mononuclear cells (PBMCs).

### Isolation of PBMCs

PBMCs from patients with T1DM or HCs were isolated from EDTA-anticoagulated blood using density gradient centrifugation with Ficoll-Paque PLUS. Briefly, blood was diluted 1:1 with PBS, layered over Ficoll, and centrifuged at 1000 × g for 30 min at room temperature. PBMCs were collected from the interface, washed twice with PBS containing 2% fetal bovine serum, and centrifugated at 3000 rpm for 10 min. The PBMC pellet was resuspended in RNA Extraction Reagent (AG, China) and stored at − 80 °C.

### RNA Extraction and Quantitative Real-Time PRC (qRT-PCR)

Total RNA was isolated from PBMCs using the AG RNAex Pro RNA Extraction Reagent (AG, China). After removing the genomic DNA, reverse transcription was performed using the Evo M-MLV Reverse Transcription Kit (AG, China). Reported A260/A280 ratios (2.0–2.1) and RNA Integrity Number (RIN, 7.5–8.5) for all samples, confirming high-quality RNA. The relative transcript abundance was determined through qRT-PCR analysis using the SYBR® Green Pro Taq HS Premixed qPCR Kit (AG, China) with *GAPDH* as the internal reference gene. Quantitative real-time polymerase chain reaction (qRT-PCR) was conducted to determine the relative transcript abundance using SYBR® Green Pro Taq HS Premixed qPCR Kit (AG, China) on a Bio-Rad CFX96 Real-Time PCR System. *GAPDH* was used as the internal reference gene. The qRT-PCR cycling conditions were as follows: initial denaturation at 95 °C for 3 min, followed by 40 cycles of denaturation at 95 °C for 10 s and annealing/extension at 60 °C for 30 s. The expression levels of target genes were calculated relative to *GAPDH* using the 2^−ΔΔCt^ method. The primer sequences are as follows (Table 1):

**Table 1:** Primer sequence for qRT-PCR

Target gene		Primer sequence (5′−3′)
*FOS*	Forward	5'- TACTACCACTCACCCGCAGA −3'
	Reverse	5'- GAAGTTGGCACTGGAGACG −3'
*JUNB*	Forward	5'- CTTCAAGGAGGAACCGCAGAC −3'
	Reverse	5'- CGCTCTTGGTCTTCCATGTTGA −3'
*DUSP1*	Forward	5'- GGACAACCACAAGGCAGACAT −3'
	Reverse	5'- TCATAAGGTAAGCAAGGCAGATGG −3'
*NR4A2*	Forward	5'- GTATGGGTCCTCGCCTCAAG −3'
	Reverse	5'- AGCCTGTGCTGTAGTTGTCC −3'
*GAPDH*	Forward	5'- GTGAAGGTCGGAGTCAACGG −3'
	Reverse	5'- GCAACAATATCCACTTTACCAGAGT −3'

### Pathway Expression and Hub Gene Correlations

To investigate the relationship between hub genes and key signaling pathways, boxplots were generated to compare the expression of pathways, such as cAMP signaling, JAK-STAT signaling, and MAPK signaling, in the HC and T1DM groups. Pearson correlation coefficients were computed to analyze the strength and direction of associations between hub genes and pathways, and a correlation heatmap was created to visualize these relationships. Correlations were considered significant when *p* < 0.05 and were denoted by an asterisk (*) in the heatmap.

### Comparative Analysis of Biological Processes

We employed validated gene set scoring systems to quantify the activity of angiogenesis, EMT, and hypoxia. These scoring systems were constructed based on previously curated gene sets specific to each process: Angiogenesis was evaluated using a gene set enriched for markers of vascular endothelial cell proliferation, migration, and tube formation (e.g., VEGFA, ANGPT1, TIE2), which are critical for new blood vessel formation [14]. EMT scoring relied on genes associated with the transition of epithelial cells to a mesenchymal phenotype (e.g., SNAI1, TWIST1, VIM), a process linked to tissue fibrosis and organ damage in diabetic complications [15]. Hypoxia was assessed using a gene set targeting hypoxia-responsive genes (e.g., HIF1A, LDHA, VEGFA), which are upregulated under low-oxygen conditions, a common feature of tissues with impaired perfusion in T1DM [16]. For each sample, a composite score was calculated by aggregating the normalized expression levels of genes in each gene set, using the single-sample Gene Set Enrichment Analysis (ssGSEA) algorithm [17]. This approach converts gene expression data into a continuous score representing the relative activity of the biological process, enabling quantitative comparisons between groups. The biological processes of angiogenesis, EMT, and hypoxia were assessed using established scoring systems. Boxplots were employed to compare the scores of these processes between the T1DM and HC groups, with significance tested using the Wilcoxon rank-sum test. A correlation heatmap was used to examine the relationships between hub genes and these processes, with significant correlations highlighted by an asterisk (*).

### Metabolite Levels and Hub Gene Correlations

To assess metabolic changes in the T1DM group, metabolite levels for metabolic score prediction were derived from METAFlux [18]. Metabolite pathway activity was predicted from gene expression data using METAFlux v2.0, which integrates genome-scale metabolic models to infer flux through biochemical reactions based on transcriptomic profiles. Key metabolites, including glucose, leucine, phenylalanine, and proline, were compared between the HC and T1DM groups using boxplots. Pearson correlation analysis was performed to examine the correlations between hub genes and metabolites, and a correlation heatmap was generated to visualize these associations. Significant correlations were marked with an asterisk (*), indicating strong associations with altered metabolic pathways in T1DM.

### Statistical Analysis

Used R v4.3.1 with packages limma (for DEG analysis), clusterProfiler (GO/KEGG enrichment), and ggplot2 (visualization). Adjusted *p*-values using the Benjamini–Hochberg method for false discovery rate (FDR) control, with a threshold of FDR < 0.05 for significant DEGs.

## Results

### Functional Enrichment Analysis of DEGs

**Fig. 1:**
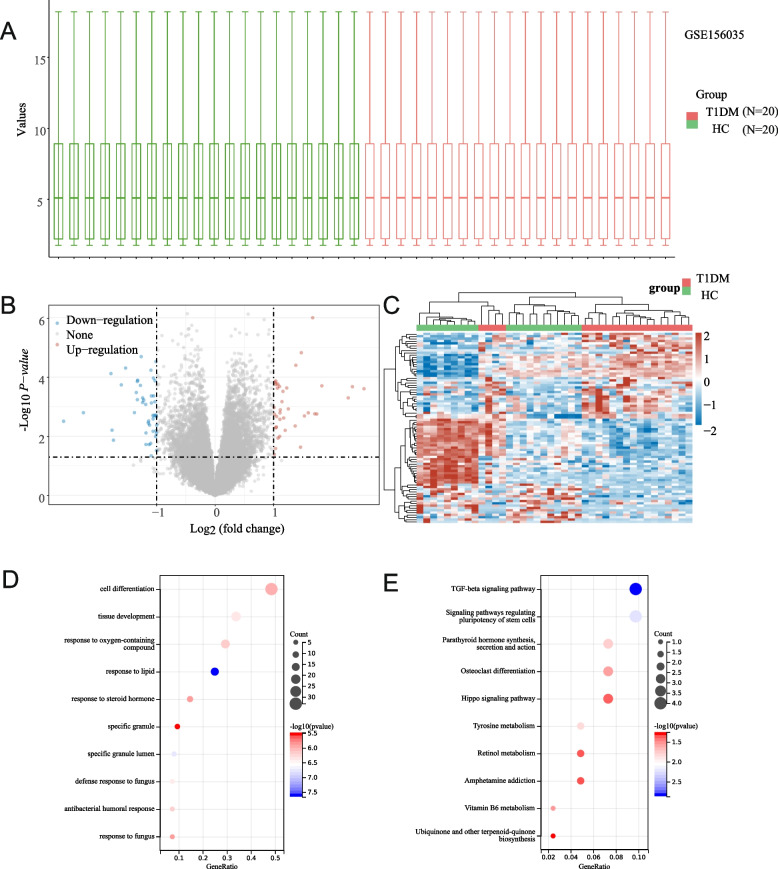
Analysis of differential gene expression, pathway enrichment, and functional annotations based on the GSE156035 dataset. **A** A boxplot demonstrating the expression levels of genes in the GSE156035 dataset across all samples, showing the normalization and distribution. **B** A volcano plot of differentially expressed genes (DEGs), with significantly upregulation genes marked in red and downregulation genes in blue. **C** A heatmap of the top DEGs, grouped by experimental conditions, highlighting clustering patterns. **D **GO enrichment analysis of DEGs. **E** KEGG pathway enrichment analysis of DEGs

The gene expression dataset GSE156035 was analyzed to identify DEGs between the HC and T1DM groups. A boxplot of normalized gene expression levels (Fig. 1A) confirmed consistent data distribution across all samples. A volcano plot (Fig. 1B) revealed substantially upregulated genes (red) and downregulated genes (blue). A heatmap of the top DEGs (Fig. 1C) demonstrated obvious clustering patterns, differentiating the HC and T1DM groups. A total of 37 upregulated genes and 45 downregulated genes were identified based on the criteria |log2 fold-change|> 1 and adjusted *p* value < 0.05. To characterize the biological annotations of the DEGs, Gene Ontology (GO) and Kyoto Encyclopedia of Genes and Genomes (KEGG) pathway enrichment analyses were systematically undertaken. GO analysis identified significant enrichment of DEGs in canonical biological processes implicated in cell differentiation, tissue morphogenesis, and cytokine-mediated signaling cascades, among other ontological categories. KEGG pathway mapping disclosed that the T cell receptor signaling axis emerged as the most prominently enriched pathway, underscoring its centrality in the autoimmune pathogenesis of T1DM. Notably, pathways governing cytokine-cytokine receptor crosstalk and Th17 cell lineage commitment were also significantly enriched, highlighting coordinated immune dysregulation at the systemic level. Concurrently, metabolic pathways related to vitamin assimilation and nutrient catabolism were implicated, reflecting the pleiotropic metabolic derangements inherent to T1DM pathophysiology.

### PPI Network and Hub Gene Identification

**Fig. 2:**
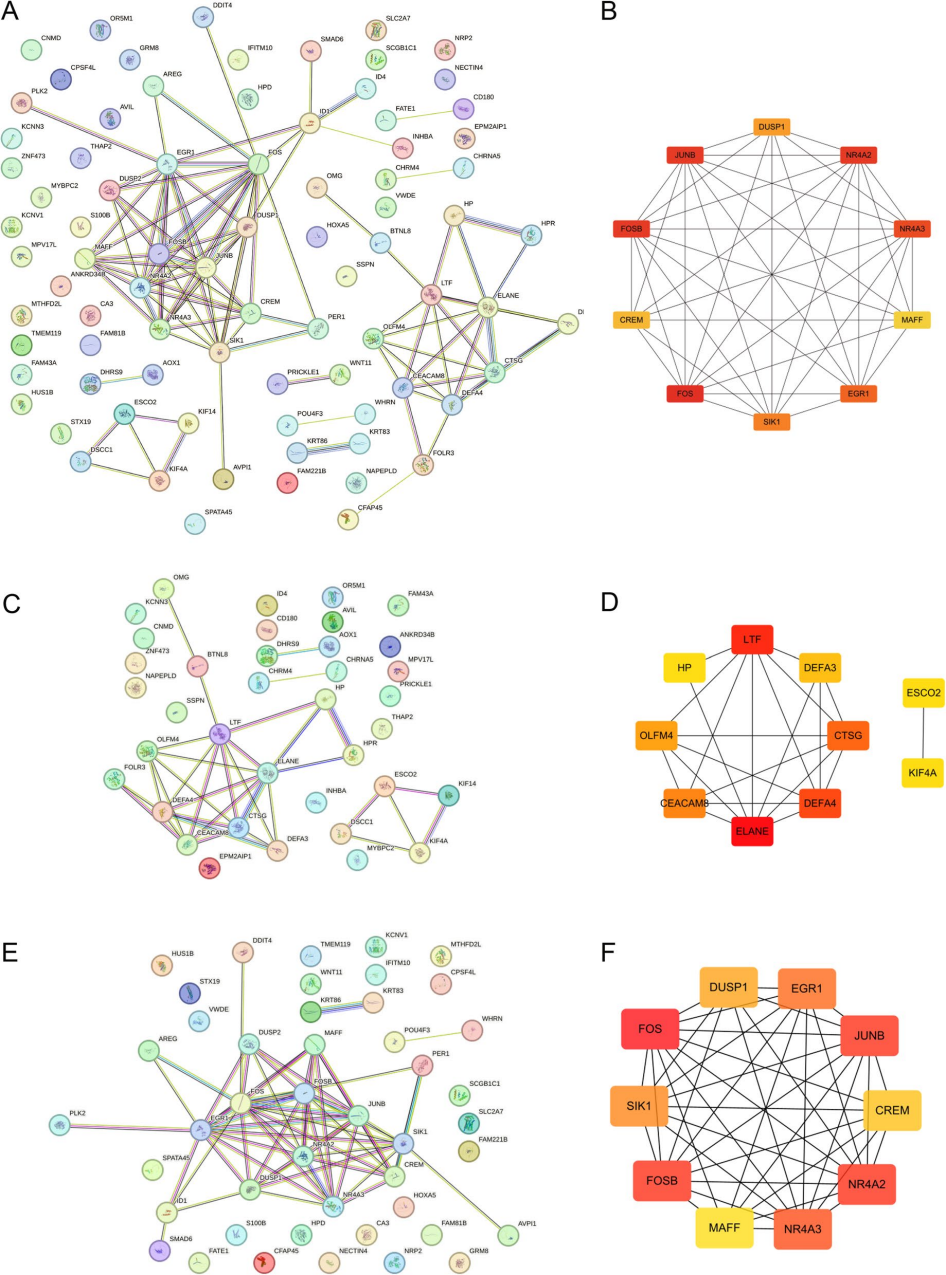
A protein–protein interaction (PPI) network and hub gene analysis of differentially expressed genes (DEGs). **A** PPI network constructed for the DEGs using the STRING database. Each node represents a gene/protein, and the connections represent interactions. The densely connected regions highlight key functional modules. **B** Hub gene analysis of the PPI network, identifying key hub genes, such as *FOS*, *JUNB*, *NR4A2*, and *DUSP1*. The node color gradient represents the degree of connectivity, with red indicating higher connectivity and importance. **C** PPI network and hub gene analysis for up-regulated genes. **D** PPI network and hub gene analysis for down-regulated genes

The DEGs were further assessed using the STRING database to construct a PPI network, which was visualized using Cytoscape (Fig. 2A). The PPI network revealed significant interactions among the DEGs, forming central functional modules. Hub gene analysis (Fig. 2B) identified critical genes, including *FOS*, *JUNB*, *NR4A2*, and *DUSP1*, based on their degree of connectivity within the network. These hub genes were prioritized as key mediators with potential roles in the pathophysiology of T1DM. In the up-regulated gene sub-network (Fig. 2D), a tightly connected cluster was observed. Key genes within this cluster include LTF and IL18-related nodes. For the down-regulated gene sub-network (Fig. 2F), core hubs centered around genes such as DUSP1 and EGR1 were identified.

### Verification of the Transcriptome-derived DEGs

**Fig. 3:**
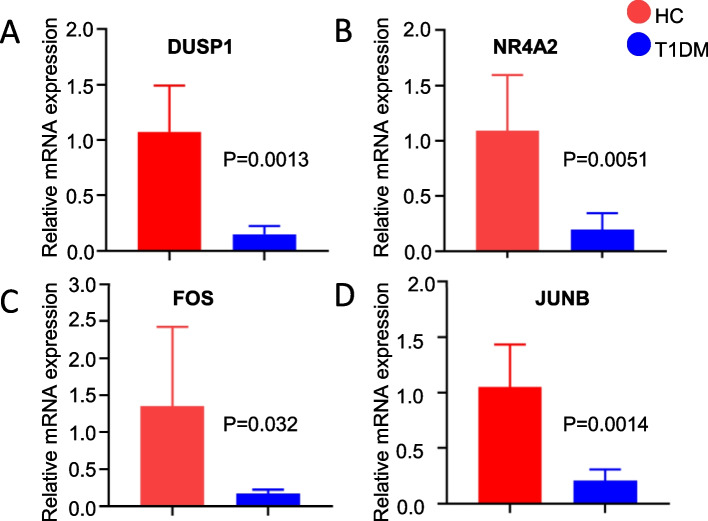
Validation of hub gene expression levels using qRT-PCR. **A** Relative mRNA expression of *DUSP1*. **B** Relative mRNA expression of *NR4A2*.** C** Relative mRNA expression of *FOS*. **D** Relative mRNA expression of *JUNB*

To further study the expression observed in the PPI network, we isolated PBMCs from the blood samples of patients and performed qRT-PCR to validate the mRNA expression levels of DEGs. As expected, the expression levels of *FOS*, *JUNB*, *NR4A2* and *DUSP1* were significantly downregulated in the PBMCs of T1DM patients as compared to the HCs (Fig. 3).

### Analysis of Pathway Expression and Hub Gene Correlations

**Fig. 4:**
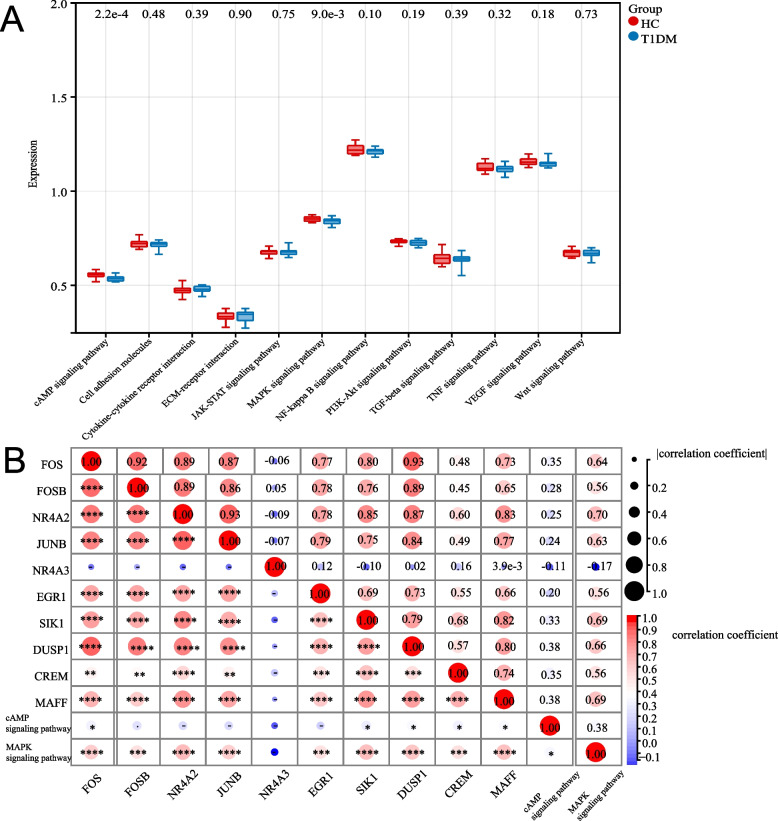
Expression and correlation analysis of key signaling pathways and hub genes in the HC and T1DM groups. **A** A boxplot comparing the expression of signaling pathways between the HC and T1DM groups. **B** A correlation heatmap illustrating the correlations between central hub genes and signaling pathways. The circle size and color intensity represent the strength and direction of the correlation, respectively, with significant correlations indicated by asterisks (*)

Central signaling pathways and their correlations with hub genes were examined in the HC and T1DM groups. Boxplots (Fig. 4A) demonstrated substantial differences in the expression of signaling pathways, such as the cAMP, JAK-STAT, and MAPK signaling pathways, with *p* values annotated above the plots. A correlation heatmap (Fig. 4B) showed strong associations between hub genes (*FOS*, *JUNB*, and *NR4A2*) and key pathways, including the cAMP and MAPK signaling pathways. The strength and direction of the correlations were visualized using circle size and color intensity, respectively, with significant correlations denoted by asterisks.

### Comparative Analysis of Biological Processes

**Fig. 5:**
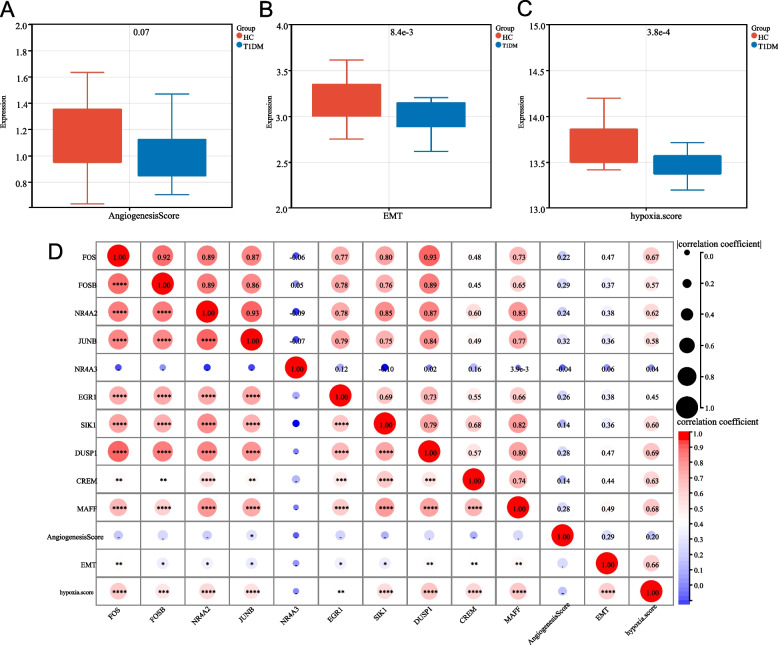
Comparative analysis of angiogenesis, epithelial-mesenchymal transition (EMT), and hypoxia scores, and their correlations with hub genes in the HC and T1DM groups. **A–C** Boxplots comparing the angiogenesis score (**A**), EMT score (**B**), and hypoxia score (**C**) between the HC and T1DM groups. Significant differences were observed in the EMT and hypoxia scores, with *p* values annotated above the plots. **D** A correlation heatmap demonstrating the correlations between hub genes (e.g., *FOS*, *JUNB*, and *NR4A2*) and biological processes, including angiogenesis, EMT, and hypoxia. The circle size and color intensity represent the strength and direction of the correlation, respectively, with significant correlations indicated by asterisks (*)

To further investigate specific biological processes, such as angiogenesis, EMT, and hypoxia, comparative analyses were performed. Boxplots (Fig. 5A–C) demonstrated considerable increases in EMT and hypoxia scores in the T1DM group compared with the HC group, with *p* values annotated above the plots. The correlation heatmap (Fig. 5D) revealed strong relationships between hub genes (*FOS*, *JUNB*, *NR4A2*) and biological processes, such as angiogenesis, EMT, and hypoxia. These findings suggest that hub genes play essential roles in mediating these processes, which are closely associated with the pathophysiology of T1DM.

### Metabolite Levels and Hub Gene Correlations

**Fig. 6:**
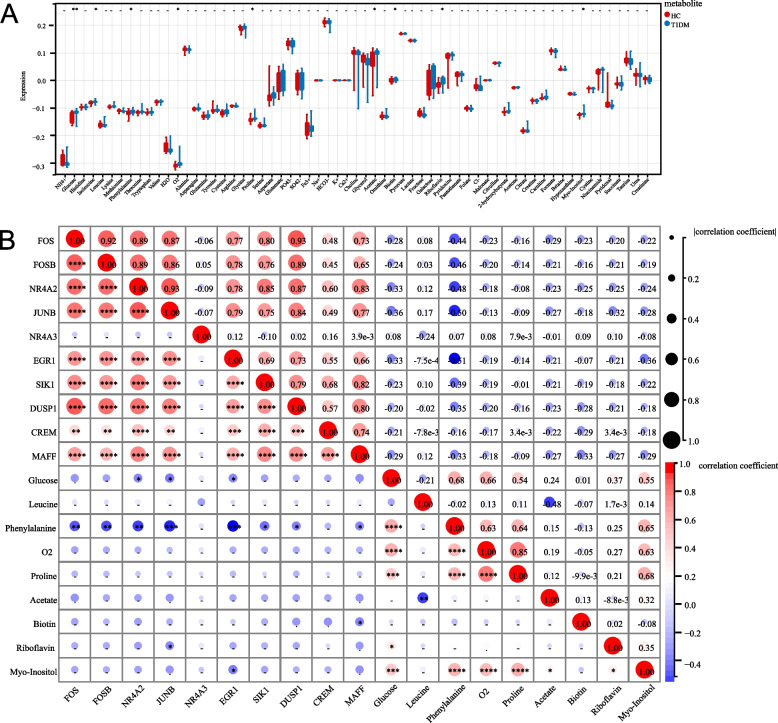
Comparative analysis of metabolite levels and their correlations with hub genes in the HC and T1DM groups. **A** A boxplot comparing the levels of various metabolites between the HC and T1DM groups. The metabolites included glucose, leucine, phenylalanine, proline, and others, with significant differences marked. **B** A correlation heatmap demonstrating the correlations between hub genes (e.g., *FOS*, *JUNB*, and *NR4A2*) and key metabolites (e.g., glucose, acetate, and myo-inositol). The color and size of the circles represent the strength and direction of the correlation, respectively, with significant correlations marked by asterisks (*)

To investigate metabolic changes in T1DM, chief metabolite levels were compared between the HC and T1DM groups, and their correlations with hub genes were analyzed. Boxplots (Fig. 6A) demonstrated considerable differences in metabolite levels, such as glucose, leucine, phenylalanine, and proline, between the two groups. A correlation heatmap (Fig. 6B) showed significant relationships between hub genes (*FOS*, *JUNB* and *NR4A2*) and key metabolites, including glucose, acetate, and myo-inositol. *FOS*, *JUNB* and *NR4A2* were positively correlated with all genes except *NR4A3* (*p* < 0.05), and *FOS*, *JUNB* and *NR4A2* were negatively correlated with phenylalanine (*p* < 0.05). Significant correlations are indicated by asterisks, indicating the strong association of these genes with altered metabolic pathways in T1DM.

## Discussion

T1DM is an autoimmune-mediated metabolic disorder characterized by the absolute deficiency of insulin secretion due to pancreatic β-cell destruction [19], with chronic hyperglycemia leading to devastating complications including retinopathy, nephropathy, and neuropathy. This condition imposes significant burdens on both patients and healthcare systems [20–22]. Despite advances in insulin replacement therapy and glucose monitoring [23, 24], current management strategies remain suboptimal, facing challenges such as glycemic variability, hypoglycemic risks, and the inability to prevent disease progression [25]. The complex interplay between genetic predisposition, immune dysregulation [26, 27], and metabolic disturbances during T1DM underscores the urgent need for a more thorough molecular characterization to identify novel therapeutic targets effective against T1DM [28].

This study used an integrative bioinformatics approach combined with experimental validation to elucidate the transcriptomic landscape of PBMCs in T1DM. Our analysis revealed significant changes in central signaling pathways and metabolic networks and specifically identified hub genes that may play a role in orchestrating disease-relevant biological processes. The following discussion will elaborate on how these findings provide mechanistic insights into the pathophysiology of T1DM, focusing on (1) the functional implications of DEGs in immune-metabolic crosstalk, (2) the central regulatory roles of identified hub genes within PPI networks, and (3) the potential clinical translation of pathway-specific interventions targeting these molecular signatures.

The identification of key DEGs in PBMCs offers crucial insights into the molecular mechanisms underlying T1DM pathogenesis [29]. The substantial downregulation of *FOS*, *JUNB*, *NR4A2*, and *DUSP1* indicates that these genes potentially play a role in β-cell dysfunction, as these genes are known regulators of cellular stress responses and apoptosis [29–32]. Functional enrichment analyses shed light on the biological processes and pathways driving these transcriptional changes. GO terms related to cell differentiation, tissue morphogenesis, and cytokine-mediated signaling cascades align with the multifaceted nature of T1DM pathophysiology. For instance, altered cell differentiation may relate to the impaired immune cell maturation observed in T1DM, where immune cells fail to maintain self-tolerance, leading to β-cell destruction [33]. The enrichment of cytokine-mediated signaling underscores the central role of proinflammatory cytokines (e.g., IL-6, TNF-α) in orchestrating autoimmune responses, as these molecules promote T cell activation and recruitment to pancreatic islets [34]. EGG pathway analysis further prioritized key molecular axes, with the T cell receptor signaling pathway emerging as the most significantly enriched. This finding is consistent with the well-established role of T cells as primary mediators of β-cell autoimmunity in T1DM [35]. Activation of the T cell receptor signaling axis triggers downstream cascades that promote T cell proliferation, differentiation into pathogenic subsets (e.g., Th1 and Th17 cells), and secretion of cytotoxic molecules, all of which contribute to β-cell damage [36]. The concurrent enrichment of cytokine-cytokine receptor crosstalk and Th17 cell lineage commitment pathways reinforces this immune dysregulation: Th17 cells, characterized by IL-17 secretion, have been implicated in pancreatic islet inflammation, while dysregulated cytokine signaling amplifies the proinflammatory milieu [37]. Notably, the enrichment of metabolic pathways related to vitamin assimilation and nutrient catabolism bridges immune and metabolic disturbances in T1DM. Vitamin metabolism, such as retinol (vitamin A) metabolism (also highlighted in the Results), is critical for immune cell function—retinoic acid, a metabolite of retinol, regulates T cell differentiation and gut immune homeostasis [38]. Disruptions in this pathway may impair immune regulation, exacerbating autoimmunity. Additionally, altered nutrient catabolism (e.g., of amino acids like leucine and phenylalanine, as observed in metabolite analyses) reflects the systemic metabolic derangements in T1DM, where insulin deficiency leads to impaired nutrient uptake and utilization. These metabolic changes may further modulate immune cell function, as immune cells rely on specific nutrients (e.g., glucose, amino acids) for activation and cytokine production, creating a feedforward loop between metabolic stress and immune activation [39].

The PPI network analysis revealed *FOS*, *JUNB*, *NR4A2* and *DUSP1* as key hubs, indicating their pivotal role in coordinating the molecular alterations observed in T1DM. The high connectivity of these genes within the network suggests that they may function as master mediators of several pathological processes. This finding is particularly significant as these hub genes have been implicated in other autoimmune conditions, which suggests common pathogenic mechanisms across autoimmune disorders [40]. The strong correlation between these hub genes and essential pathways, such as the JAK-STAT and MAPK signaling pathways, further supports the potential of these pathways as therapeutic targets. Notably, numerous existing drugs targeting these pathways (e.g., JAK inhibitors) could be repurposed for T1DM treatment. As such, further investigation into the effects of these potentially repurposed drugs on β-cell preservation and immune modulation is warranted [41].

The observed metabolic changes, specifically in glucose, leucine, and phenylalanine metabolism, coupled with their correlations to hub gene expression, provide a novel perspective on the metabolic-immune axis in T1DM. The negative correlation between *NR4A2* and glucose metabolism suggests that this nuclear receptor may play an unrecognized role in metabolic regulation in T1DM [42]. These findings bridge the gap between genetic predisposition and metabolic dysfunction in T1DM, potentially explaining how genetic variants contribute to disease susceptibility through metabolic pathways. The substantial alterations in hypoxia and EMT scores, along with their association with hub genes, may explain the early development of microvascular complications in patients with T1DM, even before overt hyperglycemia becomes apparent [43].

While this study provides valuable insights into the molecular mechanisms underlying the pathophysiology of T1DM through integrated multi-omics analyses, several limitations should be acknowledged. The current study relied on microarray data from the GSE156035 dataset to identify DEGs and explore molecular mechanisms in T1DM. While microarray technology has been widely used for gene expression profiling, it has inherent limitations that should be acknowledged. First, microarray analysis is constrained by predefined probes, which restricts the detection of novel transcripts, alternative splicing events, and low-abundance genes. Second, the dynamic range of microarrays is relatively narrow compared to RNA-seq, potentially leading to underestimation of fold changes in gene expression, especially for highly upregulated or downregulated genes. Additionally, cross-hybridization of homologous sequences may introduce noise, affecting the accuracy of DEG identification. In contrast, RNA-seq offers significant advantages, including unbiased detection of all transcripts (including unannotated ones), higher sensitivity for low-abundance genes, and a broader dynamic range for quantifying gene expression. These features are critical for dissecting the complex molecular networks in T1DM, such as identifying rare but functionally important transcripts involved in hypoxia or metabolite regulation. Future studies employing RNA-seq datasets would enable more precise characterization of transcriptomic landscapes, facilitate the discovery of novel hub genes or non-coding RNAs, and enhance our understanding of T1DM pathophysiology at the molecular level. The relatively small sample size (*n* = 10) may decrease the statistical power and generalizability of the study findings. Thus, validation of the study findings in larger, independent cohorts is necessary. Although qRT-PCR confirmed the expression trends of hub genes (*FOS*, *JUNB*, *NR4A2* and *DUSP1*), the lack of functional experiments (e.g., gene knockout or overexpression) limits the mechanistic interpretations of the roles of these genes in the pathogenesis of T1DM. Additionally, reliance on public metabolomics data introduces potential batch effects, and the absence of longitudinal clinical data prevents the assessment of the predictive value of hub genes in T1DM disease progression or complications. Future studies should address these gaps to strengthen the translational relevance of the identified targets.

## Conclusion

This study systematically identified key hub genes (*FOS*, *JUNB*, *NR4A2* and *DUSP1*) and their associated signaling pathways (e.g., MAPK and JAK-STAT) in T1DM by integrating transcriptomic, PPI network, and metabolomic analyses. The dysregulation of these genes and pathways highlights their potential roles in immune dysregulation, metabolic disturbances, and microvascular complications characteristic of T1DM. Despite limitations, our findings provide a foundation for future research aimed at developing targeted therapies. Further validation through functional assays and clinical studies will be critical to translating these molecular insights into actionable diagnostic or therapeutic strategies for T1DM.

## Data Availability

No datasets were generated or analysed during the current study.

## Abbreviations

T1DM: Type 1 diabetes mellitus
DEGs: Differentially expressed genes
EMT: Epithelial-mesenchymal transition
PPI: Protein-protein interaction
HCs: Healthy controls
GO: Gene ontology
GEO: Gene expression omnibus
PBMCs: Peripheral blood mononuclear cells
